# Predictors of blood glucose change and microvascular complications of type 2 diabetes mellitus patients in Felege Hiwot and Debre Markos referral hospital, North West Ethiopia

**DOI:** 10.1186/s12902-022-01047-x

**Published:** 2022-05-23

**Authors:** Nigusie Gashaye Shita, Ashagrie Sharew Isayu

**Affiliations:** Department of Statistics, Debre Markos University, Debre Markos, Ethiopia

**Keywords:** Type 2 diabetes mellitus, Microvascular complications, Blood glucose level, Adjusted Hazard Ratio, Survival submodel, Longitudinal submodel, Joint model

## Abstract

**Background:**

Microvascular complications lead to disability, dependency, and accelerated morbidity and mortality. This study aimed to identify predictors of blood glucose change and time to microvascular complications among patients with type 2 diabetes.

**Methods:**

A retrospective cohort study was conducted among type 2 diabetes mellitus patients enrolled between December 2014 and December 2015 at Felege Hiwot and Debre Markos Referral Hospital. A total of 318 T2DM patients were included in the study. Joint modelling of longitudinal and survival analysis was employed to identify predictors of Blood Glucose Change and Microvascular Complications in Type 2 Diabetes Mellitus Patients.

**Results:**

The prevalence of microvascular complications in Type 2 diabetes patients was 26.3%, 95%confidence interval(CI):(21.5, 31.1). Of which, half of the patients developed a microvascular complication after 30 months from the onset of the follow-up. The significant predictors of developing microvascular complication were positive proteinurea (adjusted hazard ratio (AHR) = 1.418, 95%CI: 1.080, 1.861), Serum creatinine (AHR = 3.704, 95%CI: 1.992, 6.887), Weight (AHR = 1.058, 95%CI: 1.023, 1.094), and log fasting blood glucose(log(FBS))(AHR = 1.013, 95%CI: 1.010, 1.015). The predictors of fasting blood glucose progression were higher baseline FBS(est(estimate) = 0.002,95%CI:0.0018, 0.0022), Systolic blood pressure (SBP) (est = 0.003, 95%CI: 0.002, 0.004), diastolic blood pressure (DBP) (est = 0.002, 95%CI: 0.0002, 0.004), and age (est = 0.003, 95%CI: 0.001, 0.004).

**Conclusion:**

The progression of the fasting blood glucose level for rural patients was faster than for urban patients. Patients having higher baseline FBS, previous hypertension history, higher SBP, higher DBP, older age, and fewer visits to the hospital have a relatively more progressive change in blood sugar levels. Patients having higher triglyceride levels, positive proteinuria, higher fasting blood sugar, higher weight, and a lesser number of hospital visits have a higher risk of developing a complication. In response to this finding, an aggressive intervention that targets to prevent microvascular complications is required.

## Background

Diabetes mellitus (DM) is a metabolic disorder of multiple etiologies characterized by chronic hyperglycemia with disturbances in carbohydrate, fat, and protein metabolism resulting from defects in insulin secretion, insulin action, or both [1].

Globally, the prevalence of diabetes was expected to be 8.3% (6.2–11.8) in 2019 among individuals aged 20–79, including 50.1% who are undiagnosed. It will be expected to be 10.2% (8.1–13.2) by 2030. In African Region, with 59.7%, undiagnosed diabetes has a prevalence of 3.9% (2.1–7.1) among individuals aged 20–79 and it will be projected to 4.1% (2.3–7.5) by 2030. In Ethiopia, the prevalence of diabetes was predicted to be 3.2% of individuals aged 20–79 years in 2019 [2, 3]. The international burden of disease data suggests DM may be responsible for 4.2 million individuals aged 20–79 years death in 2019 [2].

T2DM is a rapidly rising non-communicable disease and a major public health challenge in Ethiopia with the end result of disability and premature death due to long-term effects of untreated diabetes mellitus [2, 3]. Properly managing blood sugar is the most important means of effectively preventing complications associated with T2DM [4]. However, the proportion of uncontrolled levels of blood sugar in T2DM was far above the ground in Ethiopia [5–9]. Due to this, the prevalence of diabetes complications was high in Ethiopia.

For instance, around 18.8% of T2DM developed microvascular complications in the worldwide [10], and this prevalence was increased to 45% in the Middle East [11] and 47.8% in African diabetes [12]. The prevalence of diabetes microvascular complications is widespread in Gondar, Ethiopia, 20.4% [13], Wollega hospital 31.2% [14], Gurage zone 61% [15], Jimma university hospital 41.5% [16], and Mettu Karl Referral Hospital 38.5% [17].

The associated factors of the changes of FBS among type 2 diabetes mellitus patients were residence, gender, age, duration of follow-up, hypertension history, baseline FBS, number of hospital visits per follow-up, body mass index, alcohol use, diet, exercise, education status, family history, and treatment type [9, 18–20]. Until now, previous studies have not assessed the influence of SBP and DBP on the progression of FBS [9, 18–20]. Besides, most of the studies on the associated factors of FBS among T2DM were cross-sectional in Ethiopia [6, 21–24].

The predictors of microvascular complications among type 2 diabetes mellitus patients were residence, gender, age, marital status, family history of diabetes mellitus, hypertension, weight, glycemic control, medication, and adherence to diet [14, 15, 25–28]. Yet, previous studies did not assess the influence of high-density lipoprotein C, low-density lipoprotein C, and Triglyceride-C on the predictors of microvascular complications [14, 15, 25–28]. Besides, there is no evidence on how these factors are associated with microvascular complications and changes in FBS by using a joint modelling approach.

Prior studies conducted in Ethiopia used separate analyses of longitudinal and survival data by ignoring the association between longitudinal markers (FBS) and time to microvascular complications. Hence, joint modeling approaches are better than separate analysis and provide valid and efficient inferences when the longitudinal marker is correlated with the survival process, either with the subject status as well as the possibility of study dropout [29–31]. Therefore, this study aimed to identify predictors of blood glucose change and time to microvascular complications among patients with type 2 diabetes using the joint modelling approach.

## Methods

### Study design, study area, and study period

An institutional-based retrospective follow-up study design was used. Data were collected from Felege-Hiwot and Debre Markos Referral Hospitals, Northwest Ethiopia. Patients who enrolled from December 2014 to December 2015, were followed up on until January 2020. Felege Hiwot Referral Hospital is found in the capital city of Amhara Regional State, Bahir Dar city, and Debre Markos Referral Hospital is found in the head quarter of the east Gojjam zone, Debre Markos town.

### Source and study population

The source of the population was all T2DM patients who had follow-up at Felege-Hiwot and Debre Markos Referral Hospitals. The study population was all T2DM patients who had follow-up at Felege-Hiwot and Debre Markos Referral Hospitals during the study period who fulfilled the inclusion criteria of the study. A total of 318 patients and 2024 observations were included in the analysis.

### Inclusion and exclusion criteria

T2DM patients with at least two fasting blood glucose measurements within the study period, patients who had free from any of the vascular complications at the start of treatment and above the age of 18 years were included during the study, whereas the patient chart would not be available during the data collection period and patients with missing key predictor variables were excluded from the study.

### Study variables

The dependent variables were the length of time (measured in months) from the date of admission to the hospital until the date of microvascular complications development or not, and blood glucose levels in terms of fasting plasma glucose (measured in milligrams per deciliter). The independent variables were socio-demographic characteristics (age, sex, residence), clinical variables (weight, duration of DM, specific type of drug regimen, hypertension history, number of hospital visits), and physiological variables (serum creatinine, SBP, DBP, HDL-C, LDL-C, Triglyceride-C level, cholesterol and proteinuria).

### Operational definitions

HDL-C is defined as low if the HDL-C level is < 40 mg/dl and high if it is ≥ 40 [32–34].

LDL-C is defined as low if the LDL-C level is ≤ 100 and high if it is > 100 [32–34].

Hypertension comorbidity is a history of antihypertensive drug use or SBP ≥ 140 mmHg or DBP ≥ 90 mmHg [34, 35].

Total cholesterol is high if the cholesterol level is greater than 200 mg/dl and low if it is200mg/dl or lower [32–34].

Protein urea is defined as positive if the urine albumin concentration is between 30 mg/24 h and 300 mg/ 24 h and negative if it is < 30 mg/24 h.

Triglyceride-C level is low if the Triglyceride-C level is ≤ 200 and low if it is > 200 [32–34].

### Data collection methods and data quality control

Both baseline and follow-up data on socio-demographic characteristics, clinical characteristics, and physiological characteristics were collected from patient cards. In longitudinal data we have found missing values. To treat it, we have used the complete case analysis missing handling mechanism. Since, this method may be preferred under situations in which the number of observations (sample size) is large and the missing data mechanism is missing completely at random [36].

Microvascular complications such as retinopathy, nephropathy, and neuropathy were determined based on the clinical decision of the physician. Diabetic retinopathy was defined by both direct and indirect ophthalmoscope assessments done by retinal specialists confirmed by fundus photography. Neuropathy was defined by a history of numbness, paraesthesia, tingling sensation confirmed by touch sensation by 10 g monofilament, vibration sense by biothesiometer, and ankle reflex. Nephropathy was defined as worsening of blood pressure control, swelling of the foot and ankle, hands, or eyes, and increased need to urinate protein in the urine with confirmation by tests like blood test, urine test, renal function test and imaging tests [34, 37, 38].

The data were collected by two nurses who had experience with diabetic patients follow-up. To maintain data quality, training was given to the data collectors and their supervisors. The data extraction checklist was pretested for consistency of understanding of the review tools and completeness of data items. The necessary adjustments were made to the final data extraction format and the filled formats were checked daily by the supervisor.

### Ethics approval and consent to participate

Ethical approval to conduct the study and human subject research approval for this study was received from Debre Markos University, College of Natural and Computational Sciences Research Ethics Committee with reference number NCS/1620/10/011. We confirm that all methods were performed according to the relevant guidelines and regulations. Due to the retrospective nature of the study, the need for informed consent was waived according to the Research Ethics Committee of Natural and Computational Science College (Debre Markos University), but the data were anonymous and kept confidential.

### Data analysis

Descriptive statistics such as minimum, maximum, percentages, means, medians, inter-quaritile range, and standard deviation were used to describe the study population. Profile plots were used to visualize the patterns of individual profiles and average progression changes in fasting blood glucose graphically.The survival experiences of patients among different groups were estimated and compared by using Kaplan–Meier survival function and Log rank test, respectively. The significant differences in mean fasting blood glucose were assessed by using independent t-test or One-Way ANOVA. Joint models were used to identify predictors of changes in blood glucose levels and time to microvascular complications simultaneously. The joint models consist of two linked submodels, known as the longitudinal sub model and the survival sub model. From Longitudinal sub model random intercept linear mixed models with AR(1) covariance structure were used to identify predictors of the change of blood glucose level over time and from the survivable sub model log logistics parametric models were used to identify the predictors of time to microvascular complications.

### Longitudinal sub model

**Fig. 1:**
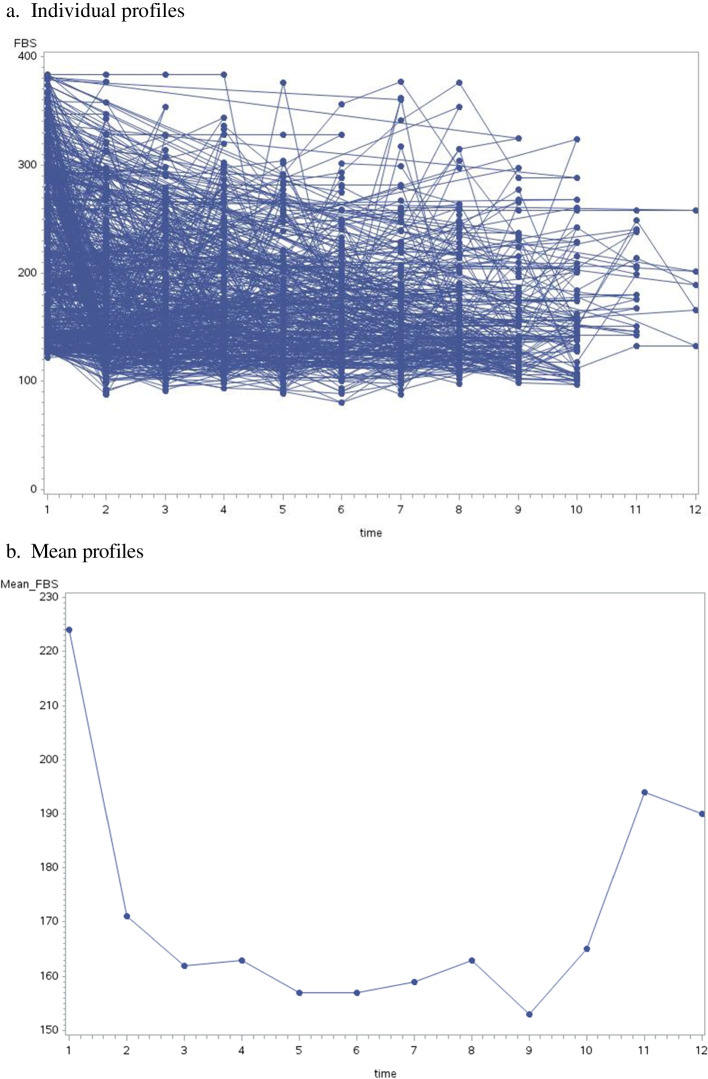
**a-b** Individual and Mean profiles for FBS levels of type 2 diabetes patients in DMRH and FHRH, December 2014-December 2020

**Fig. 2:**
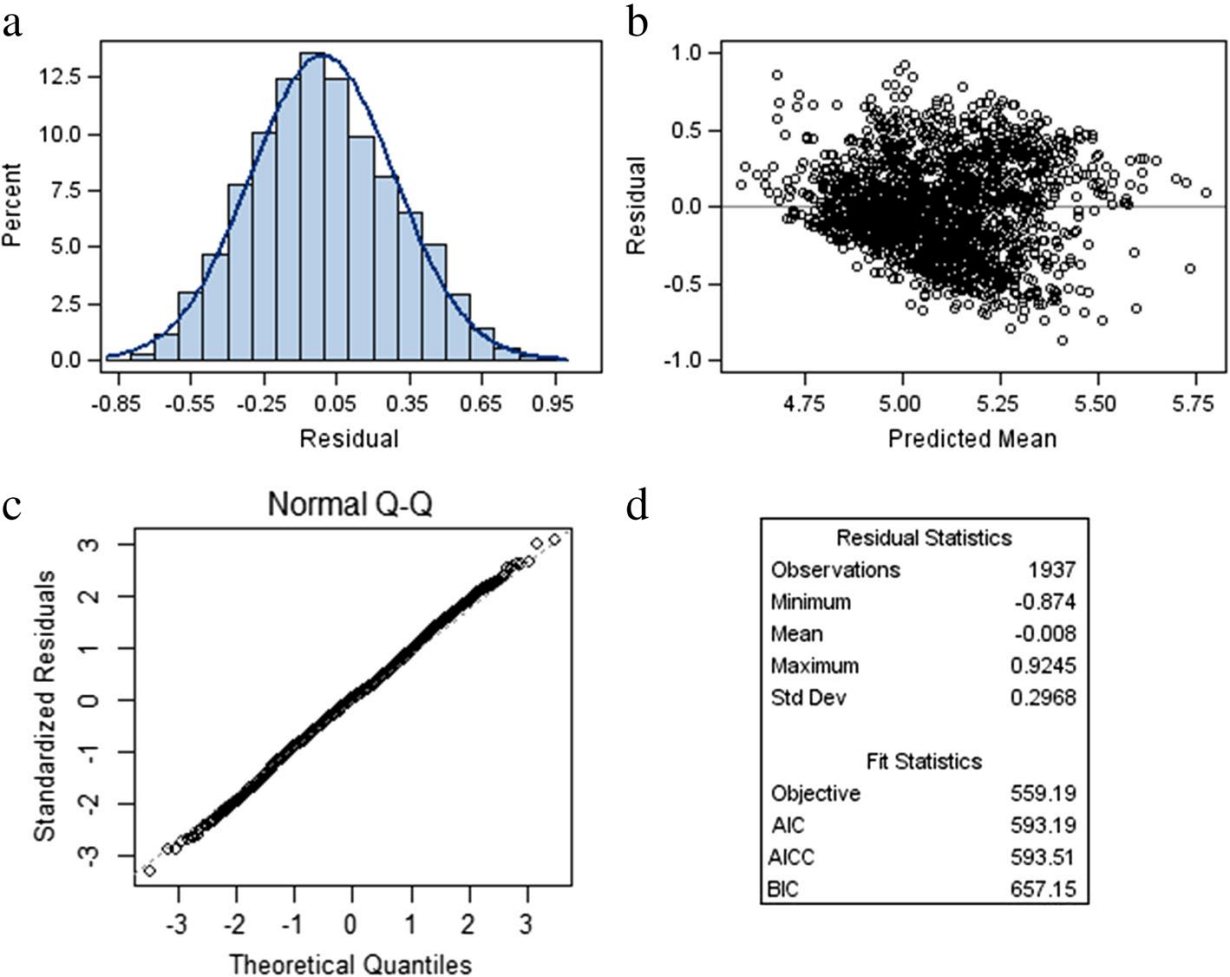
Residual plots for log Fasting plasma glucose of T2DM patients at DMRH and FHRH, December 2014-December 2020

A linear mixed submodel was used to assess the determinant factors for the progression of blood glucose levels by analyzing the repeated measures data, FBS values. The individual and mean profile plots of FBS levels for diabetic patients in Fig. 1 show that the linearity assumption is not reasonable. Therefore, we analyzed the longitudinal data structure of the FBS level by using log-transformed in the mixed model framework. Since, the transformed data of the residual plots in Fig. 2 show that both the linearity and normality assumptions meet. The linear mixed submodel can be rewritten as:

1 $$\begin{array}{c}{{\mathrm y}_{\mathrm i}\left(\mathrm t\right)=\mathrm m}_{\mathrm i}\left(\mathrm t\right)+{\mathrm u}_{\mathrm i}\left(\mathrm t\right)+\in_{\mathrm i}\left(\mathrm t\right)\\{\mathrm m}_{\mathrm i}\left(\mathrm t\right)={\mathrm x}_{\mathrm i}\left(\mathrm t\right)\mathrm\beta+{\mathrm z}_{\mathrm i}\left(\mathrm t\right){\mathrm b}_{\mathrm i}\\{\mathrm b}_{\mathrm i}\sim\mathrm N\left(0,{\mathrm\Sigma}_{\mathrm b}\right)\\\in_{\mathrm i}\sim\mathrm N(0,\mathrm\sigma^2{\mathrm I}_{\mathrm{ni}})\end{array}$$

where y is an $n\times 1$ observational of FBS values, $\beta$ is a $p\times 1$ vector of unknown constants of fixed effects of the model, $X$ is an $n\times p$ known matrix of fixed effects associated with $\beta$, Z is an $n\times q$ known design matrix of random effects,${b}_{i}$ is a $q\times 1$ vector of unknown random effects, and ${\epsilon }_{i}\left(t\right)$ is an n $\times 1$ vector of error terms. Since the FBS values taken from a patient at different follow-up times are assumed to be serially correlated, the stochastic term ${u}_{i}\left(t\right)$ is used to capture the remaining serial correlation in these observed measurements, not captured by the random effects [31]. The stochastic term is considered as a zero-mean stochastic process, independent of ${b}_{i}$ and ${\epsilon }_{i}\left(t\right)$.

Measurements made on the same subject are likely to be more similar than measurements made on different individuals. That is, repeated measures are correlated. For an analysis to be valid, the covariance among repeated measures must be modeled properly. Specifically, for the current study, the AR(1) covariance structure was used because, as we show in Table 1, the AR(1) covariance structure has the smallest AIC and BIC than other covariance structures.

**Table 1:** Summarized value of Information criteria for T2DM Patients data at DMRH and FHRH, December 2014-December 2020

Title		AIC	BIC
Parametric survival distributions	Exponential Distribution	395.1	421.4
	Weibull Distribution	354.9	388.7
	Loglogistic Distribution	354.7	381.0
	Log normal Distribution	359.0	392.8
Models for longitudinal Analysis	Marginal model	597.5	657.7
	Random intercept Linear mixed model	593.2	657.1
	Random intercept and slope Linear mixed model	599.4	663.4
Covariance structure for longitudinal Analysis	Compound symmetry	587.1	652.9
	Autoregressive order one	585.1	648.9
	Toeplitz	591.2	659.0
	Unstructured	602.2	661.0

### Survival sub model

The survival submodel was used to identify factors that affect the time taken until a T2DM patient develops some form of microvascular complication. The survival submodel has the form:

2 $$log{T}_{i}= \mu +{\alpha }_{1}{w}_{1i}+{\alpha }_{2}{w}_{2i}+\cdots +{\alpha }_{p}{w}_{pi}{+\theta m}_{i}\left(t\right)+\sigma {\epsilon }_{i}$$

where $\mu$ is the intercept, ${w}_{ji}$ denote the j^th^ baseline covariate of the i^th^ observation with a corresponding vector of regression coefficients ${\alpha }_{j}(j=\mathrm{1,2},\cdots \cdots \cdots ,p)$, ${T}_{i}$ denotes the observed failure time for the i^th^ subject $(i=\mathrm{1,2},\cdots \cdots \cdots ,n)$,${m}_{i}\left(t\right)$ is the unobserved value of the longitudinal outcome at the time $t$, $\sigma$ is the scale parameter, and ${\epsilon }_{i}$ denote the i^th^ observation error terms having a standard probability distribution. Specifically, for this study, the log-logistic distribution is an appropriate probability distribution than others. Since, as we have shown in Table 1, the loglogistic distribution has the smallest AIC and BIC than other probability distributions.

### Parameter estimation for joint modelling

The joint model parameters were estimated by using the restricted maximum likelihood estimation method. In addition, we have used a pseudo-adaptive Gauss Hermite numerical integration method to get the approximate solution for the joint model parameters.

To build both separate longitudinal and survival analyses, first, we fit a univariable model for each of the explanatory variables and, based on statistical significance, identified the variables to be candidates for the multivariable analysis. As naturally different factors/variables do not operate separately, multivariable analysis helps to control for confounders and analyze the effects of a factor in the presence of other factors in the model.

After we have applied the above model-building strategies, longitudinal and survival submodels were fitted by joining the separated longitudinal and survival analyses using a JM package of R 3.4. We used Akaike and Bayesian information criteria to select the appropriate joint models, and the model with the smallest AIC or BIC was considered the best fit [39, 40].

## Results

### Characteristics of study participants

Out of the total of 318 newly diagnosed type 2 DM patients, 193 (60.7%) were males. The mean (± SD) age of patients was 55.19(± 11.55) years. The majority of the patients, 227(71.4%) were urban dwellers. More than half of the patients, 177(55.7%) had hypertension at the start of type 2 DM treatment. About 243(76.4%) were with one oral agent user. Less than one-fourth of the patients, 77(24.2%) had positive protein urea at baseline. Less than one-fifth 42(13.2%) of type 2 DM patients included in the study had triglyceride levels ≤ 150 mg/dl. The mean (± SD) for SBP and DBP of the patients was 128(± 17.6) and 79.7 (± 11.2) respectively (Table 2). The median values for serum creatinine and FBS were found to be 1.26 mg/dl (IQR = 1.04–1.3) and 223.83 mg/dl (IQR = 149–292.25) respectively.

**Table 2:** Population characteristics and Univariate associations of factors with log of fasting blood glucose at DMRH and FHRH, December 2014- March 2020

Variables	Categories	Total (% Any one of Microvascular complication)	Mean	Std. Dev	Minimum	Maximum	*P* value
Gender	Female	125(28.0%)	176	65	88	381	0.025
	Male	193(35.2%)	170	63	80	384	
Residence	Rural	91(35.2%)	190	70	80	381	0.000
	Urban	227(31.3%)	167	61	88	384	
Age	Continuous	-	55.19	11.55	25	80	0.016
Hypertension history	Yes	177(29.9%)	177	67	80	384	0.015
	No	141(35.5%)	169	61	89	384	
Weight	Continuous	-	72.04	6.31	58	87	0.515
HDL-C(mg/dl)	< 40	212(37.3%)	168	62	80	384	0.000
	≥ 40	106(22.6%)	182	67	89	381	
LDL-C(mg/dl)	≤ 100	209(35.9)	169	62	80	384	0.000
	> 100	109(25.7%)	180	68	89	381	
Triglyceride(mg/dl)	≤ 150	42(57.1%)	169	60	92	381	0.549
	> 150	276(28.6%)	173	65	80	384	
Cholesterol(mg/dl)	≤ 200	277(32.1%)	172	64	80	384	0.99
	> 200	41(34.1%)	173	65	80	381	
Protein urea	Positive	77(35.2%)	172	64	80	384	0.916
	Negative	241(31.5%)	173	64	90	381	
Treatment	one oral agent	243(24.7%)	181	65	88	376	0.000
	more than one oral agent (no insulin)	43(32.1%)	178	65	89	384	
	insulin alone or insulin plus oral agents	32(34.6%)	168	63	80	384	
Serum creatine	Continuous	-	1.18	0.26	0.24	3.47	0.983
SBP	Continuous	-	128.0	17.6	80	200	0.000
DBP	Continuous	-	79.7	11.2	40	130	0.000
Number of visit	Count	-	21.9	13.2	2	48	0.000

### Microvascular complications among T2DM patients

T2DM patients were followed for a median follow-up period of 30 months (IQR = 13–44.7) after initiation of treatment (Table 3).

**Table 3:** Summary statistics of continuous variables included in the study of TDM patients at DMRH and FHRH, December 2014- March 2020

Status of patient	Continuous variables	Mean	Median	Q1	Q3	Standard deviation	Minimum	Maximum
Any one of Micro vascular Complication	Time	27.96	26	14	39.53	13.39	3.1	65
	Age	52	52	47.5	60	9.66	28	80
	no _visit	17	11.5	6	22	10.07	3	45
	Creatinine	1.21	1.26	1.14	1.3	0.19	0.7	1.63
	weight	73.75	72	72	76	5.68	58	87
	SBP	127	130	120	130	17.72	90	180
	DBP	79	80	70	90	12.93	40	120
	FBS	224	233.5	156.5	295.5	74.36	126	384
No events	Time	29.8	37.93	12.63	47.17	18.67	3	66
	Age	56	56	45	65	12.34	25	80
	no _visit	17	15	5	30	14.08	3	48
	Creatinine	1.14	1.26	1	1.26	0.24	0.24	1.82
	weight	72.29	72	71	73	4.04	58	85
	SBP	132	130	120	140	20.9	90	200
	DBP	82	80	70	90	12.89	50	120
	FBS	224	205.5	147	292	77.08	126	384
Over all	Time	29.22	30	13	44.7	17.69	3	66

**Fig. 3:**
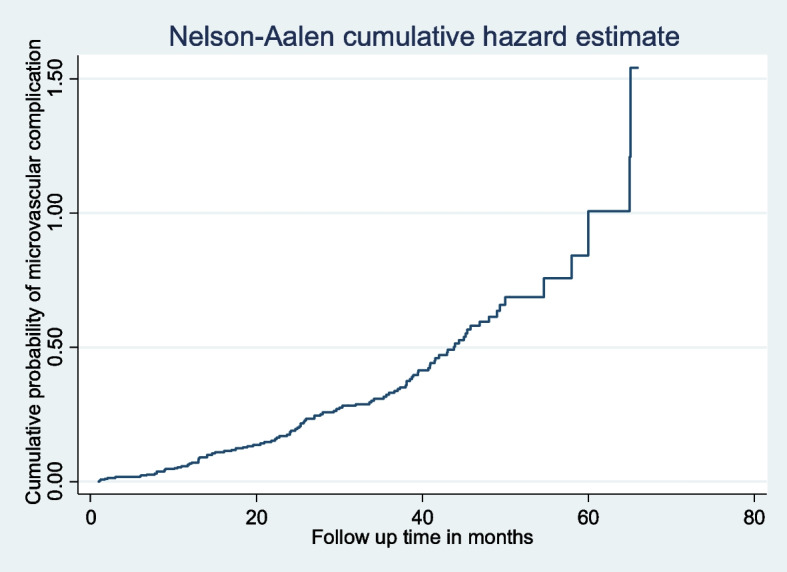
The Nelson-Aalen cumulative hazard estimated plot of type 2 diabetes patients in DMRH and FHRH, December 2014-December 2020

During the study period, the incidence of microvascular complications was 32.4 cases (95% CI: 27.2–37.5) per100 person-year observation. From this, the incidence of retinopathy was 9.1 (95%CI:6.0–12.3), nephropathy was 7.5(95%CI:4.6–10.5), and neuropathy was 15.7cases(95%CI: 11.7–19.7) per100 person-year observation. The cumulative probability of developing microvascular complications among type 2 DM patients was 0.0497at month 10, 0.14234at month 20, 0.276 at month 30, 0.42443 at month 40 and 0.69034 at month 50 (Fig. 3).

### Predictors of fasting blood glucose among T2DM patients

The minimum measurement taken on the subject was two times, while the maximum measurement taken on the subject was 23 repeated times. The minimum and maximum fasting blood glucose of the patients was 80 and 384 mg/dl, respectively, under the study period. Female T2DM patients had higher mean fasting blood glucose values (176 mg/dl) than male T2DM patients(170 mg/dl).

Patients with HDL-C levels ≥ 40 mg/dl had higher mean fasting blood glucose values (182 mg/dl) than those with HDL-C levels < 40 mg/dl (168 mg/dl). Patients with LDL-C levels > 100 mg/dl had higher mean fasting blood glucose values (180 mg/dl) than those with LDL-C levels ≤ 100 mg/dl (169 mg/dl). Insulin alone or insulin plus oral agent users had higher mean fasting blood glucose values (181 mg/dl) than multiple oral medication users (178 mg/dl) and that one oral agent user (168 mg/dl) (Table 2).

After multivariable analysis using the longitudinal submodel, the variable number of hospital visits, residence, baseline FBS, hypertension history, SBP, DBP, and age were predictors of the change in FBS among type 2 DM patients.

Urban T2DM patients had a 0.06 mg/dl lower FBS level compared to those rural residence T2DM patients. The expected FBS was increased by 0.0027 mg/dl when the age of the patients increased by one year by keeping constant other covariates.

The expected FBS level decreased by 0.0032 mg/dl when the number of hospital visits per follow-up period increased by one day by keeping constant covariates. Patients who had no hypertension history had a 0.04 mg/dl lower FBS level compared to those who had hypertension history.

As baseline FBS increased by one mg/dl, the expected FBS level was increased by 0.002 mg/dl given that the other covariates are constant.

As SBP and DBP increased by one mmHg, the expected FBS level was increased by 0.0029 mg/dl and 0.002 mg/dl, respectively, by controlling other covariates (Table 5).

### Predictors of microvascular complications among T2DM patients

**Fig. 4:**
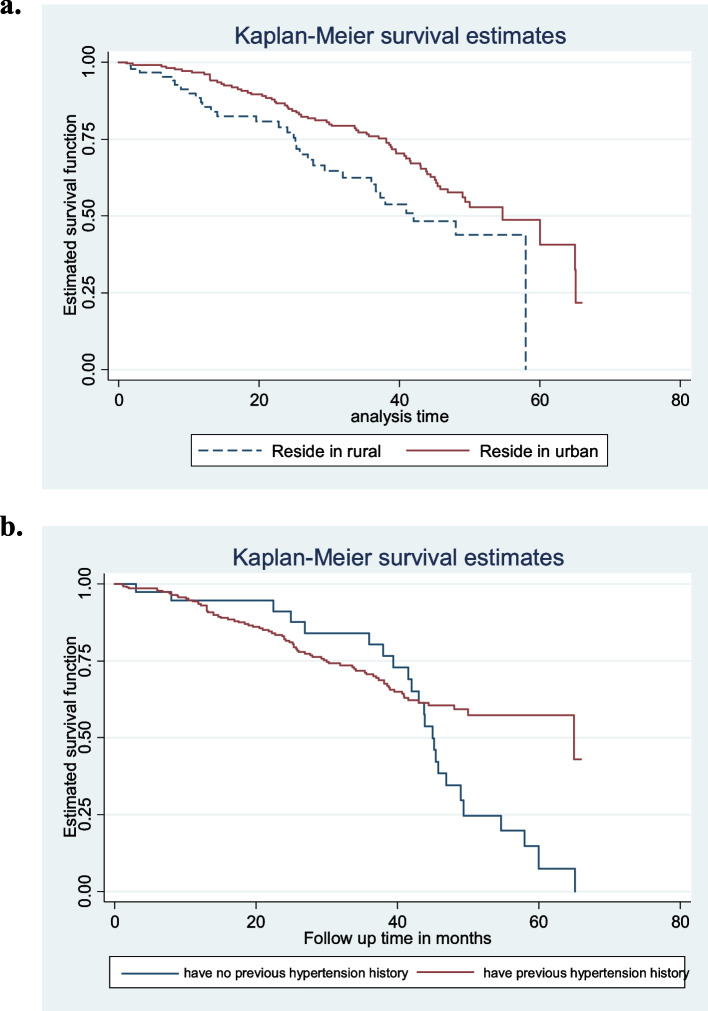
**a-b** Plots of Kaplan–Meier survivor functions for categorical variables of type 2 diabetes patients in DMRH and FHRH, December 2014-December 2020

The survival experiences of patients who had no previous hypertension history and resided in urban areas were significantly greater than those of patients with previous hypertension history and resided in rural areas, respectively (Table 4, Fig. 4).

**Table 4:** Results of the Log-rank test for the categorical variables of T2DM patients at DMRH and FHRH, December 2014-December 2020

Covariate/factor	DF	Chi-square	*P*-value
Gender	1	2.32	0.128
Residence	1	5.98	0.0145
Hypertension	1	8.64	0.003
Protein urea	1	4.89	0.027
HDL-C(mg/dl)	1	0.641	0.423
LDL-C(mg/dl)	1	0.347	0.556
Triglyceride(mg/dl)	1	5.048	0.025
Cholesterol(mg/dl)	1	0.047	0.829
Treatment	2	1.893	0.388

After multivariable analysis using the survival submodel, the variable number of hospital visits, serum creatine, triglyceride level, proteinuria, weight, and unobserved fasting blood glucose were predictors of microvascular complications among type 2 DM patients.

The risk of developing microvascular complications was increased by 1.06 months when the weight of the patients increased by one kg, while the hazard of developing microvascular complications was decreased by 0.95 months when the number of hospital visits per follow-up period increased by one day.

The hazard of developing a microvascular complication for positive proteinuria was increased by 1.42 months compared with that of negative proteinuria patients. Patients with triglycerided ≤ 150 mg/dl had 0.47 months’ lower hazard of developing microvascular complications compared to those patients with triglycerided > 150 mg/dl.The risk of developing microvascular complications was increased by 3.7 months when serum creatinine of the patients increased by one mg/dl. The hazard of developing microvascular complications increased by 1.01 months when the true unobserved log fasting blood glucose increased by one mg/dl (Table 5).

**Table 5:** Result of Longitudinal and Survival Sub model onT2DM patients at DMRH and FHRH, December 2014-December 2020

	Variable	Estimate	Standard error	95% confidence level		*P* value
				LCL	UCL	
Longitudinal process	Intercept	4.5766	0.1718	4.2399	4.9133	< 0.0001
	Time	-0.0284	0.0036	-0.0355	-0.0213	< 0.0001
	Residence Urban	-0.0597	0.0213	-0.1014	-0.0180	0.0051
	Residence Rural(ref)	-	-	-	-	-
	Number of hospital visits	-0.0032	0.0007	-0.0046	-0.0018	< 0.0001
	Baseline FBS	0.002	0.0001	0.0018	0.0022	< 0.0001
	Hypertension history No	-0.0446	0.0196	-0.0830	-0.0062	0.0231
	Hypertension history (ref)	-	-	-	-	-
	SBP	0.0029	0.0006	0.0017	0.0041	< 0.0001
	DBP	0.0020	0.0009	0.0002	0.0038	0.0275
	Age	0.0027	0.0008	0.0011	0.0043	0.001
	Weight	-0.0001	0.0021	-0.0042	0.0040	0.9437
	Identification of patients	0.0069	0.0028	0.0015	0.0122	0.0063
	Autoregressive order one	0.3015	0.0349	0.2331	0.3699	< .0001
	Residual	0.0808	0.0038	0.0734	0.0882	< .0001
Event process	Intercept	8.2803	1.4111	5.5145	11.0461	< 0.0001
	Number of hospital visits	0.0551	0.0077	0.0400	0.0702	< 0.0001
	Serum creatinine	-1.3095	0.3164	-1.9296	-0.6894	< 0.0001
	Triglyceride-C(mg/dl) ≤ 150	0.7589	0.3476	0.0776	1.4402	0.029
	Triglyceride-C > 150 (ref)	-	-	-	-	-
	Proteinuria Positive	-0.3492	0.1387	-0.6211	-0.0773	0.0118
	Proteinuria Negative (Ref)					
	Weight	-0.0560	0.0171	-0.0895	-0.0225	0.001
	Association	-0.0126	0.0012	-0.0150	-0.0102	< 0.0001
	log(shape)	0.5612	0.0961	0.3728	0.7496	< 0.0001

Formula Calculating change in FBS for longitudinal process = (${e}^{estimate}$-1), Formula calculating Adjusted hazard ratio for event process $={e}^{-estimate}$, ref = reference group

## Discussion

In this study, survival longitudinal submodel analysis was used to identify the determinant factors for the time to develop microvascular complications and changes in the blood glucose level. The study revealed that the rate of change in FBS levels in diabetic patients, due to clinic interventions, does not continue at a steady pace but changes with time, residence, number of hospital visits, baseline FBS, patients with hypertension history, age, and SBP of patients. On the other hand, the variable number of hospital visits, serum creatinine, proteinuria, triglyceride level, protineinuria, weight, and FBS were found to have a significant association with the risk of developing microvascular complications.

In this study, the overall incidence of microvascular type 2 diabetic complications was 32.4%. This proportion is in line with studies in Ghana, 35.3% [41] and Wollega, Ethiopia, 31.2% [14]. However, this finding is higher than studies in Gondar, Ethiopia, 20.4% [13]. On the other hand, this result is lower than Metu, Ethiopia, 38.5% [17]; Gurage, Ethiopia, 61% [15]; India 69% [25]; China 57.5% [26]; Dessie, Ethiopia, 37.9% [28]. The difference might be related to sample size, follow-up time, patient’s adherence to medication, and practice of life style recommendations.

Patients who resided in urban areas had a 0.06 mg/dl lower FBS level compared to those who live in rural areas. The results of this study were similar to the study conducted in Ethiopia [18] and contradicted with other studies conducted in Ethiopia, which reported no significant association between residents with fasting blood glucose [8, 42]. The possible justification for this study might be due to lower awareness on treatment adherence in T2DM patients who live in rural areas [43]. Besides, the majority of patients may have lower educated levels.

Per year increase in the age of T2DM patients, the expected FBS increases by 0.0027 mg/dl. This finding agrees with the findings from India [44] and Ethiopia [8], but this result counteradcted with another study conducted in Ethiopia [9, 18]. The possible reasons for this study were due to the occurrence of diabetes-related complications within higher ages [45]. This implies that older age not only increases the risk of chronic illness, the management of illnesses also becomes difficult.

Per one mmHg increase in the SBP and DBP of T2DM patients, the expected FBS increases by 0.0029 mg/dl and 0.002 mg/dl, respectively. This study was consistent with the results of previous studies [46, 47]. The possible mechanism is that people with high blood pressure usually have insulin resistance and have an increased risk of developing diabetes compared to those with typical blood pressure.

Patients in the absence of hypertension history at baseline had a 0.04 mg/dl lower FBS level compared to those who had hypertension history. This finding agrees with findings from Ethiopia [9], Egypt [48], and China [49]. This is because the additional antihypertensive pill burden and complications inhibit the utilization of peripheral glucose, which finally increases the blood glucose level [50].

Per one day increase in the number of hospital visits per follow-up period of T2DM patients, the FBS decreases by 0.0032 mg/dl. Likewise, per one day increase in the number of hospital visits per follow-up period of T2DM patients, the hazard of microvascular complications decreases by 0.95. This finding was in line with previously conducted research from Ethiopia [9]. The possible reason is that the recovery process was better among patients who regularly visited a doctor [51].

In this study, higher weights were found to increase the risk of microvascular complications. It was similar to the study done in Gurage, Ethiopia [15] and China [52]. The possible justification is that the higher weight will be increased blood sugar and increased fat composition [53].

T2DM patients with hypertension comorbidity had lower survival experiences than non-hypertensive diabetes. This is similar to findings from Mettu, Ethiopia [17], Dessie, Ethiopia [28], and China [26]. The possible justification is that hypertension has a direct effect on retinal endothelial cells and function that causes cell growth and vasoconstriction, which eventually predisposes patients to microvascular complications [41].

Excess levels of triglycerides above the normal range have increased the hazard of microvascular complications, which might be due to producing plaque in the arteries and increased accumulation of fat [54].

The current study revealed that patients with positive protein urea and higher serum creatinine have an increased risk of having microvascular complications, which might be due to the fact that protein urea and serum creatine are an early sign of kidney damage. For this reason, patients with a positive protein urea and higher serum creatinine are at increased risk of microvascular complications like nephropathy in the long run [9, 54].

The risk of microvascular complications rises with growing fasting blood glucose levels. This is in line with a study done from Ethiopia [9] and from Asia, Australasia, Europe, and North America [55]. The possible reason is that augmented blood glucose levels lead to damage to retinal blood vessels and glomeruli [56].

This study expected that all microvascular complications were caused by type 2 diabetes mellitus. This may overestimate the prevalence of microvascular complications in T2DM patients. Besides, the limitation of this study was that data on some potential important predictors, such as the type of intervention were not available, which may have influenced the outcome variables.

## Conclusions and recommendations

The prevalence of microvascular complications in Type 2 Diabetes patients in this study was 32.4%. Half of the patients in the study developed any form of microvascular complication after 30 months from the onset of the follow-up time**.** The progression of the fasting blood glucose level of rural T2DM patients was faster than urban T2DM patients. Patients who had no previous hypertension history had a lower progression change in FBS compared to those patients with previous hypertension history.

Patients having higher baseline FBS, higher SBP, higher DBP, higher age, and fewer visits to the hospital had a relatively more progressive change in blood sugar levels.

Patients having higher triglyceride levels, positive proteinuria with higher fasting blood sugar, higher weight, and a fewer number of hospital visits have a higher risk of developing a complication. In light of these findings, health professionals in the DM follow-up clinics should give targeted intervention for type 2 DM patients with positive proteinuria, patients with triglycerides ≥ 150 mg/dl, with higher serum creatinine levels and higher fasting blood glucose levels to maximize effort on the prevention of T2DM complication and risk minimization of vascular complication.

## Data Availability

The data that support the findings of this study are available from Debre Markos and Felege-Hiwot Referral Hospitals, but restrictions apply to the availability of these data, which were used under license for the current study, and so are not publicly available. Data are, however available from the authors upon reasonable request and with the permission of Debre Markos and Felege-Hiwot Referral Hospitals.

## Abbreviations

AHR: Adjusted hazard ratio
AIC: Akaike information criteria
ANOVA: Analysis of variance
BIC: Bayesian information criteria
CI: Confidence interval
DBP: Diastolic blood pressure
DF: Degrees of freedom
DMRH: Debre Markos Referral Hospital
FBS: Fasting blood glucose
FHRH: Felege Hiwot Referral Hospital
HDL-C: High-density lipoprotein cholesterol
LDL-C: Low-density lipoprotein cholesterol
LCL: Lower Confidence limit
*P*-value: Probability value
Q1: Quartile one
Q3: Quartile three
SBP: Systolic blood pressure
Std. dev: Standard deviation
T2DM: Type 2 Diabetes Mellitus
UCL: Upper confidence limit
